# Status of adjuvant endocrine therapy for breast cancer

**DOI:** 10.1186/bcr3636

**Published:** 2014-04-01

**Authors:** Gaia Schiavon, Ian E Smith

**Affiliations:** 1 The Royal Marsden NHS Foundation Trust, Breast Unit, Fulham Road, London SW3 6JJ, UK; 2 The Institute of Cancer Research, 237 Fulham Road, London SW3 6JB, UK

## Abstract

Adjuvant endocrine therapy reduces the risk of recurrence and death from breast cancer in women with hormone receptor-positive early breast cancer. Tamoxifen has been the standard therapy for decades, and this is still the case for pre-menopausal women. Ovarian suppression is of similar efficacy but currently there is no strong evidence for adding this to tamoxifen and the additional morbidity can be considerable. Results from two important trials addressing this issue are imminent. In post-menopausal women, aromatase inhibitors (AIs) (letrozole, anastrozole, or exemestane) are superior to tamoxifen in preventing recurrence but only letrozole has been shown to improve survival. The main gain is against high-risk cancers, and tamoxifen gives very similar benefit for low-risk disease. Traditionally, treatment has been given for around 5 years, but many women remain at risk of relapse for 10 years or more. The AIs, and more recently tamoxifen, have been shown to reduce further the risk of late recurrence in women still in remission after 5 years of tamoxifen if given for a further 5 years. The comparative benefits of these two options and the selection of patients most likely to benefit from long-term adjuvant endocrine therapy are important topics for further research, as is the optimum duration of AI therapy started upfront.

## Introduction

**Figure 1:**
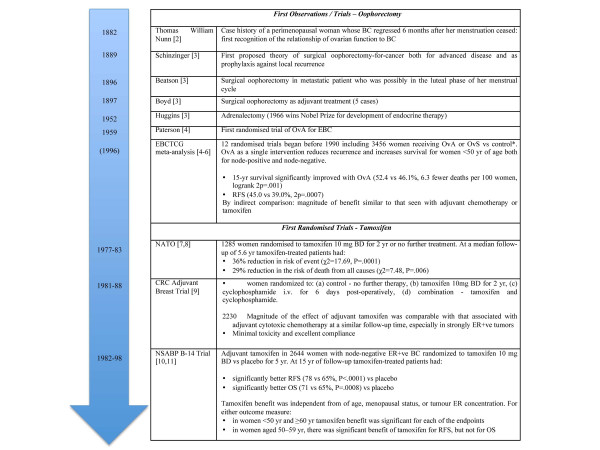
**Adjuvant endocrine therapy history: the first key steps.** The first steps in the evolution of endocrine therapy are presented. *In seven of these trials, the ovarian ablation (OvA) and control groups received no routine cytotoxic chemotherapy, in one there were random assignments both for cytotoxic therapy and for OvA in a ‘factorial’ design, and in four both groups were scheduled to receive a common cytotoxic chemotherapy regimen (after OvA, in those allocated this treatment). BC, breast cancer; BD, *bis die* (twice daily); CRC, Cancer Research Campaign; EBC, early breast cancer; EBCTCG, Early Breast Cancer Trialists’ Collaborative Group; ER, estrogen receptor; i.v., intravenously; NATO, Nolvadex Adjuvant Trial Organization; NSABP, National Surgical Adjuvant Breast and Bowel Project; OS, overall survival; OvS, ovarian suppression; RFS, recurrence-free survival; yr, year (s).

Adjuvant endocrine therapy, usually today with tamoxifen or an aromatase inhibitor (AI), is standard treatment for estrogen receptor-positive (ER^+^), early-stage breast cancer (BC), which accounts for approximately 75% of BC [1]. This is by far the oldest effective systemic treatment for any cancer, and Figure 1 illustrates the evolution of endocrine therapy, starting with Thomas William Nunn in the 1880s [2].

## First trials: oophorectomy

Oophorectomy - or ovarian ablation (OvA) - was the first form of systemic treatment for BC [3]. Although the interpretation of many trials testing OvA is limited by the small sample size or study design, their combined analysis (using age as a surrogate for menopausal status) through the Early Breast Cancer Trialists’ Collaborative Group (EBCTCG) has unequivocally established that OvA as a single intervention reduces recurrence and increases survival for women younger than 50 years of age for both axillary node-positive and node-negative disease [4]. By indirect comparison, the magnitude of the benefit was similar to that seen with adjuvant chemotherapy or tamoxifen (see below) [5,6].

## Adjuvant tamoxifen

### First trials

Results of the first randomized trials - Nolvadex Adjuvant Trial Organization, Cancer Research Campaign Adjuvant Breast Trial, National Surgical Adjuvant Breast and Bowel Project (NSABP) B-14 - showing the benefit of tamoxifen as adjuvant treatment in early BC are shown in Figure 1[7-11].

### Tamoxifen and the Oxford overview

The first Oxford EBCTCG meta-analysis involved almost 30,000 women in 28 trials with either node-positive or node-negative BC who were randomly assigned to tamoxifen (or not) for about 5 years [12]. It demonstrated a clear reduction in mortality in women at least 50 years of age treated with tamoxifen (*P* <0.0001) and a reduction in the annual odds of death during the first 5 years of about 20%. Subsequent analyses showed that the proportional risk reductions produced by tamoxifen were little affected by entry age or nodal status [13].

Long-term follow-up has greatly strengthened these findings. The most recent meta-analysis with a median follow-up of 13 years showed that, in ER^+^ disease, tamoxifen for about 5 years achieved a reduction of yearly BC mortality of about a third throughout the first 15 years (rate ratio (RR) 0.70, *P* <0.00001). The RRs were 0.53 in years 0 to 4 and 0.68 in years 5 to 9, and there was no subsequent loss of the gains made during the first decade. Over all time periods, the recurrence rate reduction averaged 39% (RR = 0.61 for any recurrence, and 0.62 for contralateral disease incidence; both two-sided *P* <0.00001) [14]. The relapse curves did not converge after year 10 (RR = 0.97 in years 10 to 14); therefore, a high proportion of patients receiving tamoxifen for 5 years can be potentially cured.

#### Tamoxifen and age, menopausal status, nodal status, size, and grade

BC mortality was significantly reduced by tamoxifen in each age group with 15-year gains of 10.6%, 4.6%, 11.7%, and 17.4%, respectively, in the ages at entry of less than 45, 45 to 54, 55 to 69, and ≥70 years. Nodal status, tumor grade, and diameter did not materially affect proportional risk reductions, but they were of course predictive of the absolute risk without tamoxifen and hence of the absolute benefit. Local recurrence, contralateral BC (generally new primary), and distant recurrence were all substantially reduced by tamoxifen (each *P* <0.00001).

#### Tamoxifen with chemotherapy

There were highly significant recurrence reductions both in the six trials with no chemotherapy (RR = 0.56) and in the 14 trials of chemotherapy plus tamoxifen versus the same chemotherapy alone (RR = 0.67), and there was a slightly greater effect of tamoxifen in those with greater degrees of ER positivity in both trial categories [13]. Even if chemotherapy was given, tamoxifen was of substantial further benefit (that is, chemotherapy plus tamoxifen was better than chemotherapy alone), producing a further reduction of about a quarter in 10-year recurrence risk, whether it was started concurrently with the chemotherapy (RR = 0.62) or after it (RR = 0.71). The slight superiority of starting concurrently was, however, not significant, and these tamoxifen trials did not randomize timing. In all regimens, tamoxifen had a substantial effect.

#### Significance of progesterone receptor

ER and progesterone receptor (PgR) status were strongly associated. PgR (when measured) was positive in 76% of ER^+^ and only 21% of ER^−^ (strictly, ER-poor) disease. Patients with ER^+^PgR^−^ disease had just as good a proportional benefit with tamoxifen (RR = 0.60) as those with ER^+^PgR^+^ (RR = 0.63) (both 2*P* <0.00001). The absolute recurrence reduction at 15 years seemed, if anything, greater in ER^+^PgR-poor than in ER^+^PgR^+^ disease, perhaps because of the higher background risk of recurrence without treatment.

The RR was 0.90 for ER^−^PgR^+^ disease (2*P* = 0.35). As assays improve, progressively fewer BCs are reported as ER^−^PgR^+^ where repeat testing on another tissue sample has been recommended, and it is likely that many of these are artefactual.

#### Tamoxifen in ER-negative and ER-low breast cancer

Tamoxifen was of no benefit where ER measurement was zero (RR = 0.97 for any recurrence; RR = 0.94 for contralateral disease). However, tamoxifen was beneficial at ER concentrations as low as 10 fmol/mg of cytosol protein with improving efficacy at increasing ER measurements. Recent guidelines suggest an immunohistochemistry cutoff of 1% to define a tumor as ER^+^[15].

#### Duration: 5 years or less

Both direct [13] and indirect [14] comparisons showed greater mortality reduction with approximately 5 versus 2 years of tamoxifen. Reductions in recurrence and mortality during years 0 to 4 were almost as great for shorter treatment duration but were less during years 5 to 9. Although the combined effects of patient drop-out and drop-in cannot be quantified exactly, the RR for BC death of 0.70 in the meta-analyses of outcome by allocated treatment suggests that in ER^+^ disease full compliance with 5 years of tamoxifen would reduce 15-year BC mortality rates by at least a third and probably more. The important issue of tamoxifen for more than 5 years is discussed below (‘Extended adjuvant endocrine therapy’).

#### Toxicities

Tamoxifen and the AIs (see below) are generally well tolerated with a low incidence of serious adverse effects (SAEs). The two serious toxicities with tamoxifen are endometrial cancer and thromboembolic events [16]. The increased uterine cancer incidence has an RR of 2.40 (*P* = 0.00002) without significant effect on other cancers [14].

The uterine cancer risk was strongly correlated with age, with little absolute risk for entry age of less than 45 years or 45 to 54 years. For entry age of 55 to 69 years, 15-year incidence rates were 3.8% in the tamoxifen group and 1.1% in the control group (absolute increase of 2.6%). In ER^+^ disease, there were nine deaths in the tamoxifen group and one in the control group from uterine cancer and six versus no deaths from pulmonary embolus (PE) during the first 5 years (but no apparent excess afterwards). A non-significant excess of stroke deaths (3 extra per 1,000 women during the first 15 years, none of which occurred during the treatment period) was balanced by a non-significant shortfall in cardiac deaths (3 fewer per 1,000 women during the first 15 years); so little net effect on overall vascular mortality was recorded. For entry age of younger than 45 years, intercurrent mortality was low, there were no deaths from uterine cancer or PE in either group, and 15-year gains in overall and BC mortality were similar.

#### CYP2D6 genotyping and tamoxifen efficacy

Tamoxifen is metabolized by the cytochrome P450 2D6 (CYP2D6) to 4-hydroxy tamoxifen and 4-hydroxy-N-desmethyl tamoxifen (endoxifen), the latter metabolite being the more abundant and more potent in terms of ER-binding affinity and suppression of estradiol-stimulated cell proliferation [17,18].

Therefore, CYP2D6-mediated metabolism is the rate-limiting enzymatic step for the formation of endoxifen, and the association of known genetic and drug factors influencing CYP2D6 enzyme activity with tamoxifen efficacy has been investigated by several groups but with conflicting results [19-24]. Recently, however, two large randomized trials - Arimidex, Tamoxifen, Alone or in Combination (ATAC) and Breast International Group (BIG) 1-98 - have shown that CYP2D6 variant alleles do not influence outcome on tamoxifen [21,22,25], and it is our view that there is no clinical indication for measuring these alleles in patients about to receive tamoxifen.

## Adjuvant ovarian suppression

The 2005 Oxford Overview (but not the most recent in 2011) included 7,601 women (age of less than 50 years) treated with either OvA (approximately 55%) or ovarian suppression (OvS) with the luteinizing hormone-releasing hormone (LHRH) (that is, goserelin) analogue, confirming a reduction of approximately 30% in recurrence and BC mortality [13]. The real benefit may be greater because many of the women in these trials had unknown receptor status. The risk reduction for women younger than 40 years was not significantly different from those who were 40 to 49. There was no significant difference in efficacy between OvA and OvS, despite a slight trend against the LHRH analogues. The benefit of OvA was sustained for up to 15 years, and an absolute difference in recurrence rate was 4.3%. This questions whether relatively short LHRH analogue treatment used today for no more than 2 to 3 years is as effective as permanent ablation.

A more detailed systematic review focused on 16 adjuvant randomized trials using LHRH agonists in 9,022 patients with hormone receptor-positive (HR^+^) BC (91.8% were ER^+^ and the remainder were ER^−^PgR^+^) [26]. In these trials, women were randomly assigned to receive an LHRH agonist or not, and other comparisons were based on chemotherapy or tamoxifen. Statistically significant reduction of recurrence (by 13%, *P* = 0.02) or death (by 15%, *P* = 0.03) after recurrence was observed when LHRH was added to agonists to tamoxifen or chemotherapy (or both) but not when used as the only systemic adjuvant treatment.

The relative merits of adjuvant OvS, tamoxifen, or the two treatments combined are still controversial. A US Intergroup trial (INT 0101) randomly assigned 1,503 pre-menopausal women pre-treated with chemotherapy to control arm (no adjuvant endocrine therapy), 5 years of goserelin, or 5 years of goserelin plus tamoxifen [27]. This showed a trend toward improvement in overall survival (OS) for goserelin versus control (hazard ratio = 0.88, *P* = 0.14) and a greater benefit for combined goserelin plus tamoxifen versus goserelin (disease-free survival (DFS): hazard ratio = 0.74, *P* <0.01 and OS: hazard ratio = 0.91, *P* = 0.21), suggesting that there may be a benefit of adding tamoxifen to goserelin on DFS but not OS.

The Austrian Breast and Colorectal Cancer Study Group (ABSCG)-12, a randomized controlled multicenter trial in 1,803 pre-menopausal women with HR^+^ BC (all receiving adjuvant goserelin), compared the efficacy and safety of anastrozole or tamoxifen with or without zoledronic acid for 3 years [28]. Of note, these patients did not receive any adjuvant chemotherapy, although approximately 5% of patients received neoadjuvant chemotherapy (balanced in the four arms). In regard to the comparison of anastrozole versus tamoxifen, the DFS was not different between the two arms, but patients on anastrozole alone had shorter OS (hazard ratio = 1.75, *P* = 0.02) at a median follow-up of 62 months (range of 0 to 114.4 months) [29]. Body mass index had a significant impact on the efficacy of anastrozole plus goserelin in these women [30]. The authors commented that incomplete suppression of estrogen production in peripheral body fat could be the cause of the reduced effect of anastrozole in the overweight and obese groups of patients. Unfortunately, the important clinical question of whether there was any gain in adding goserelin to tamoxifen was not addressed in any of the above-mentioned trials.

Long-term follow-up (median of 12 years) of 2,706 women enrolled in the Zoladex In Pre-menopausal Patients trial showed no significant difference between 2 years of tamoxifen treatment versus 2 years of goserelin versus 2 years of combined tamoxifen plus goserelin in reducing the risk for an event (recurrence, new tumor, or death) (RR = 29%, 33%, and 35%, respectively, compared with no endocrine therapy) [31]. The *P* values for the test of interaction between goserelin and tamoxifen were 0.01 (any event), 0.13 (death from any cause), 0.016 (BC recurrence), and 0.17 (death from BC).

Two important ongoing randomized phase 3 trials - Suppression of Ovarian Function Trial (SOFT) and Tamoxifen and Exemestane Trial (TEXT) - are evaluating the addition of OvS to tamoxifen and also the role of AIs in pre-menopausal women with ER^+^ early BC. SOFT compares tamoxifen versus OvS plus tamoxifen versus OvS plus exemestane for 5 years. OvS can be achieved with gonadotropin-releasing hormone analog (triptorelin) for 5 years, oophorectomy or ovarian irradiation. TEXT compares 5 years of OvS (triptorelin) plus tamoxifen to OvS (triptorelin) plus exemestane. Primary analyses for both trials are expected in late 2014 to early 2015.

In summary, current data suggest that OvS is equivalent to tamoxifen for patients in whom the latter is contraindicated, but so far there is no conclusive evidence that OvS in addition to tamoxifen, or indeed to chemotherapy, is of superior benefit, and for many women this treatment can have an adverse effect on quality of life.

## Adjuvant aromatase inhibitor trials

The development of inhibitors of aromatase, the enzyme that synthesizes estrogens from androgens, has provided an alternative strategy to deprive breast tumors of stimulation by endogenous estrogens in post-menopausal women whose ovaries are no longer active and in pre-menopausal women in whom ovarian function has been suppressed or the ovaries have been removed [32,33]. It is important to note that AIs are ineffective in pre-menopausal women with functioning ovaries [33].

Aminoglutethimide (AG) was the first AI to be developed for clinical use [34-36] and showed benefit initially in advanced disease and then as adjuvant therapy [37], but it also suppressed aldosterone and had toxicities, including rash and somnolence. A randomized clinical trial involving 2,021 post-menopausal women receiving tamoxifen alone for 5 years or in combination with AG for the first 2 years of treatment showed no significant difference in 5-year DFS and OS [38]. Moreover, more patients failed to complete combination treatment (13.7%) because of side effects compared with tamoxifen alone (5.2%, *P* = 0.0001).

Today three third-generation AIs are approved for use: anastrozole and letrozole are non-steroidal AIs that reversibly and non-covalently bind aromatase [39], and exemestane is a steroidal AI that irreversibly and covalently binds aromatase. All third-generation compounds approach nearly complete suppression of total-body aromatization and plasma estrogen levels [40,41]. In a recent study, letrozole was found to inhibit whole-body aromatization by greater than 99% in all 12 patients [41]. Third-generation AIs have been studied as adjuvant therapy against tamoxifen in a series of randomized clinical trials in post-menopausal women, both as frontline treatment and after tamoxifen (Tables 1 and 2).

**Table 1:** Main prospective, randomized, phase III clinical trials testing adjuvant aromatase inhibitors

**Study**	**Design**	**Arms**	**Number**	**Population (post-menopausal women)**	**Primary endpoint (s)**
Monotherapy (versus tamoxifen)					
ATAC [42]	Double-blind	A versus T versus T + A (5 years)	9,366	HR^+^ EBC	DFS^a^, occurrence of AEs
BIG 1-98 [43]	Double-blind	L versus T versus L→T versus T→L (5 years)	8,010	HR^+^ EBC	DFS^b^
TEAM [44,45]	Open-label, multinational	Upfront T versus E (2.75 years)	9,775	HR^+^ EBC	DFS^c^
		Sequential T→E versus E (5 years)			
Sequential therapy					
IES [46]	Double-blind	T→E versus T→T (5 years)	4,724	HR^+^ EBC	DFS^d^
ARNO 95 [47]	Open-label	T (2 years) → earsabears) versus T (2 years) → T (3 years)	979	HR^+^ EBC who received 2 years of T	DFS^a^
ABCSG Trial 8 [48]	Open-label	T (5 years) versus T (2 years)→A (3 years)	3,714	HR^+^ EBC who received 2-3 years of T	RFS^e^
ITA [49]	Open-label, multi-center	T (2-3 years)→A (5 years) versus T (5 years)	448	HR^+^ (or unknown) node^+^ EBC who received 2-3 years of T	RFS^f^
BIG 1-98 [43]	Double-blind	L versus T versus L→T versus T→L (5 years)	8,010	HR^+^ EBC	DFS^b^
TEAM [44]	Open-label, multinational	Upfront E (2.75 years) versus T	9,779	HR^+^ EBC	DFS^c^
		E (5 years) versus sequential T→E			
Extended therapy					
MA.17 [50]	Double-blind	L versus placebo	5,187	HR^+^ EBC who had received 4.5 to 6 years of adjuvant T therapy	DFS^g^
ABCSG Trial 6a [51]	Open-label	A (3 years) versus no further treatment	856	HR^+^ EBC who had received 5 years of adjuvant T, with or without AG, for the first 2 years of therapy	RFS^h^
NSABP-33 [52]	Double-blind	E (5 years) versus placebo (5 years)	1,598	HR^+^ T1-3N1M0 EBC who were disease-free after 5 years of adjuvant T	DFS^a^

^a^Time from random assignment to the occurrence of local or distant recurrence, new contralateral breast cancer, or death from any cause; ^b^time from random assignment to the first of the following events: invasive recurrence in local, regional, or distant sites; a new invasive cancer in the contralateral breast; any second (non-breast) primary cancer; or death without a previous cancer event; ^c^times from random assignment to the earliest documentation of disease relapse (locoregional or distant tumor recurrence or ipsilateral or contralateral breast cancer) or death from any cause; ^d^time from random assignment to local or distant breast cancer recurrence, new primary breast cancer, or death without recurrence (intercurrent death); ^e^time from random assignment to the earliest occurrence of local or distant recurrence or death as a result of any cause; ^f^time from random assignment to disease recurrence, including both locoregional and distant recurrences (except contralateral breast cancer); ^g^time from random assignment to the recurrence of the primary disease (in the breast, chest wall, or nodal or metastatic sites) or the development of a new primary breast cancer in the contralateral breast; ^h^interval between the start of treatment or of the observation period and the first evidence of locoregional recurrence, contralateral breast cancer, or distant metastasis. →, switch to; A, anastrozole; ABCSG, Austrian Breast and Colorectal Cancer Study Group; AE, adverse event; AG, aminoglutethimide; ARNO 95, Arimidex-Nolvadex 95; ATAC, Arimidex, Tamoxifen, Alone or in Combination; BIG, Breast International Group; DFS, disease-free survival; E, exemestane; EBC, early breast cancer; HR, hormone receptor; IES, Intergroup Exemestane Study; ITA, Italian Tamoxifen Anastrozole (trial); L, letrozole; NSABP, National Surgical Adjuvant Breast and Bowel Project; RFS, recurrence-free survival; T, tamoxifen; TEAM, Tamoxifen Exemestane Adjuvant Multinational.

**Table 2:** Outcome results in the main phase III clinical trials testing adjuvant aromatase inhibitors

**Study**	**Arms**	**DFS hazard ratio (95% CI)**	**TTR or RFS hazard ratio (95% CI)**	**TTDR or DRFI or DRFS or DDFS hazard ratio (95% CI)**	**BCFI or BCFS hazard ratio (95% CI)**	**OS hazard ratio (95% CI)**
Monotherapy analysis (versus tamoxifen)						
ATAC [53] 120-month follow-up	A versus T versus T + A (5 years)	0.91 (0.83-0.99) *P* = 0.04	TTR 0.84 (0.75-0.93) *P* = 0.001	TTDR 0.87 (0.77-0.99) *P* = 0.03	NA	0.97 (0.88-1.08) *P* = 0.6
		HR^+^ patients 0.86 (0.78-0.95) *P* = 0.003	HR^+^ patients 0.79 (0.70-0.89) *P* = 0.0002	HR^+^ patients 0.85 (0.73-0.98) *P* = 0.02		HR^+^ patients 0.95 (0.84-1.06) *P* = 0.4
BIG 1-98 [54] 8.1-year follow-up	L versus T	0.53 (0.78-0.96) *P* = 0.007	NA	DRFI 0.86 (0.74-0.998) *P* = 0.047	BCFI 0.86 (0.76-0.98) *P* = 0.03	0.87 (0.77-0.999) *P* = 0.048
		IPCW 0.82 (0.74-0.92) *P* <0.0002		IPCW 0.79 (0.68-0.92) *P* = 0.003	IPCW 0.80 (0.70-0.92) *P* = 0.002	IPCW 0.79 (0.69-0.90) *P* <0.0006
TEAM [45] 2.75-year follow-up (before the switch)	Upfront E (2.75 years) versus T	0.89 (0.77-1.03) *P* = 0.12	NA	NA	NA	NA
Sequential therapy analysis						
IES [46] 91-month follow-up	T→ E versus T→ T (5 years)	0.81 (0.72-0.91) *P* <0.001	NA	TTDR 0.84 (0.73-0.97) *P* = 0.01	BCFS 0.81 (0.71-0.92) *P* <0.001	0.53 (0.75-0.99) *P* <0.04
ARNO 95 [47] 30.1-month follow-up	T (2 years)→A (3 years) versus T (2 years)→T (3 years)	0.66 (0.44-1.00) *P* = 0.049	NA	NA	NA	0.53 (0.28-0.99) *P* = 0.045
ABCSG Trial 8 [48] 60-month follow-up	T (2 years)→A (3 years) versus T (5 years)	0.91 (0.75-1.103) *P* = 0.33	RFS 0.80 (0.631-1.013) *P* = 0.06	DRFS 0.78 (0.60-0.99) *P* = 0.046	NA	0.87 (0.64-1.16) *P* = 0.33
ITA [49] 128-month follow-up	T (2-3 years)→A (5 years) versus T (5 years)	NA	RFS 0.64 (0.44-0.94) *P* = 0.02	NA	BCFS 0.72 (0.44-1.17) *P* = 0.2	0.79 (0.52-1.21) *P* = 0.3
BIG 1-98 [54] 8.1-year follow-up	L→T versus T→L (5 years)	L→T 1.06 (0.91-1.23) *P* = 0.48	NA	L→T DRFI 1.14 (0.92-1.42) *P* = 0.24	L→T BCFI 1.10 (0.91-1.32) *P* = 0.34	L→T 0.97 (0.80-1.19) *P* = 0.79
		T→L 1.07 (0.92-1.25) *P* = 0.36		T→L 1.23 (0.99-1.53) *P* = 0.06	T→L 1.16 (0.96-1.40) *P* = 0.12	T→L 1.10 (0.90-1.33) *P* = 0.36
TEAM [55] 5-year follow-up (after the switch)	E (5 years) versus sequential T→E	1.06 (0.91-1.24) *P* = 0.42	RFS 1.06 (0.88-1.28) *P* = 0.53	NA	NA	1.00 (0.89-1.14) *P* >0.99
Extended therapy analysis						
MA.17 [56] 64-month follow-up	L versus placebo	0.68 (0.56-0.83) *P* <0.001	NA	DDFS 0.81 (0.63-1.04) *P* = 0.09	NA	0.99 (0.79-1.24) *P* = 0.83
		IPCW 0.52 (0.45-0.61) *P* <0.001		IPCW 0.51 (0.42-0.61) *P* <0.001		IPCW 0.61 (0.52-0.71) *P* <0.001
		SCC 0.58 (0.47-0.72) *P* <0.001		SCC 0.68 (0.52-0.88) *P* = 0.004		SCC 0.76 (0.60-0.96) *P* = 0.02
ABCSG Trial 6 [51] 62.3-month follow-up	A (3 years) versus no further treatment	NA	RFS 0.62 (0.40-0.96) *P* = 0.031	DFRS 0.53 (0.29-0.96) *P* = 0.034	NA	0.89 (0.59-1.34) *P* = 0.57
NSABP-33 [52] 30-month follow-up	E (5 years) versus placebo (5 years)	0.68 *P* = 0.07	RFS 0.44 *P* = 0.004	NA	NA	NA

→, switch to; A, anastrozole; ABCSG, Austrian Breast and Colorectal Cancer Study Group; ARNO 95, Arimidex-Nolvadex 95; ATAC, Arimidex, Tamoxifen, Alone or in Combination; BCFI, breast cancer-free interval; BCFS, breast cancer-free survival; BIG, Breast International Group; CI, confidence interval; DDFS, distant disease-free survival; DFS, disease-free survival; DRFI, distant relapse-free interval; DRFS, distant relapse-free survival; E, exemestane; HR^+^, hormone receptor-positive; IPCW, Inverse probability of censoring weighted; IES, Intergroup Exemestane Study; ITA, Italian Tamoxifen Anastrozole (trial); L, letrozole; NA, not available; NSABP, National Surgical Adjuvant Breast and Bowel Project; OS, overall survival; RFS, recurrence-free survival; SCC, approach proposed by Shao and colleagues [57]; T, tamoxifen; TEAM, Tamoxifen Exemestane Adjuvant Multinational; TTDR, time to distant relapse; TTR, time to relapse.

### Arimidex, tamoxifen, alone or in combination

The ATAC trial was the first trial to present data comparing adjuvant tamoxifen with an AI, and its results heralded a major change in the endocrine therapy of post-menopausal women. Tamoxifen was compared with anastrozole alone or with anastrozole plus tamoxifen for 5 years in 9,366 post-menopausal women, of whom 7,839 (84%) were known to be HR^+^[42]. At a median follow-up of 33.3 months, rates of 3-year DFS of 89.4% for anastrozole and 87.4% for the tamoxifen alone (hazard ratio = 0.83, *P* = 0.013) were seen. The combination showed no significant difference to tamoxifen alone (87.2%, hazard ratio = 1.02, *P* = 0.8). The DFS improvement with anastrozole was seen in HR^+^ but not in HR^−^ patients. The incidence of contralateral BC was significantly lower with anastrozole than with tamoxifen (odds ratio 0.42, *P* = 0.007).

After a median follow-up of 120 months, the long-term superior efficacy and safety of anastrozole over tamoxifen as initial adjuvant therapy were confirmed [53]. There were significant improvements (both in the whole cohort and in the HR^+^ subgroup) in the anastrozole group compared with the tamoxifen group for DFS, time to recurrence (TTR), and time to distant recurrence (TTDR) (Table 2). In HR^+^ patients, absolute differences in TTR between anastrozole and tamoxifen increased over time (2.7% at 5 years and 4.3% at 10 years) and recurrence rates remained significantly lower on anastrozole than tamoxifen after treatment completion (hazard ratio = 0.81, *P* = 0.03), although the carryover benefit was smaller after 8 years. There was, however, no significant difference in OS (hazard ratio = 0.95, *P* = 0.4) or in deaths after recurrence between anastrozole and tamoxifen.

### Breast international group 1-98

The BIG 1-98 study involved the other third-generation non-steroidal AI, letrozole, and compared 5 years of monotherapy with tamoxifen or with letrozole or with sequences of 2 years of one of these agents followed by 3 years of the other. The primary core analysis included all 8,010 patients but did not include any events after the first 2 years (the time of the switch) for patients in the two sequential arms [43]. These results showed that letrozole improved DFS and TTDR versus tamoxifen alone. After a median follow-up of 25.8 months, 5-year DFS estimates were 84.0% and 81.4%, respectively. Compared with tamoxifen, letrozole significantly reduced the risk of a DFS event (hazard ratio = 0.81, *P* = 0.003) and the risk of distant recurrence (hazard ratio = 0.73, *P* = 0.001).

The OS analysis of this trial was problematic as patients on the tamoxifen-alone arm were given the option to cross over to letrozole once initial results became available. Different analytical tools were developed to overcome this, including inverse probability of censoring weighted (IPCW) analysis, which achieves better estimates of relative treatment effects in the presence of selective crossover [58,59]. At a median follow-up of 8.7 years from random assignment, letrozole monotherapy was confirmed to be significantly better than tamoxifen, not just for DFS but (in contrast to ATAC) for OS by both intention to treat (ITT) and IPCW analysis (Table 2) [54].

### Sequential aromatase inhibitor treatment after tamoxifen

Several trials - including Intergroup Exemestane Study (IES), Arimidex-Nolvadex 95, ABCSG-8, and the Italian Tamoxifen Anastrozole trial - have addressed the issue of switching to an AI after 2 to 3 years of tamoxifen in post-menopausal women with ER^+^ disease [46-49]. These have consistently shown benefit for the switch and indeed tended to have lower hazard ratios for DFS than direct upfront comparisons of ATAC and BIG 1-98 (Tables 1 and 2). This led to the hypothesis that perhaps a greater gain might be achieved by starting with tamoxifen and switching rather than starting with an AI.

However, this comparison was made directly through random assignment in both the BIG 1-98 and the Tamoxifen Exemestane Adjuvant Multinational (TEAM) (see below) trials and neither has shown any basis for this hypothesis. In BIG 1-98, at a median follow-up of 8.0 years from random assignment, there was no significant difference between one of the crossover arms (tamoxifen followed by letrozole) versus letrozole alone, but there was a trend against starting with tamoxifen and then switching (DFS hazard ratio = 1.07; OS hazard ratio = 1.10; both *P* = 0.36; Table 2) [54].

The multicenter TEAM trial, originally designed to examine the efficacy of exemestane versus tamoxifen in 9,779 HR^+^ women [44], was revised in 2004, and patients on tamoxifen were switched to exemestane after 2.5 to 3 years, when the IES (Table 1) reported superior results for a switch from tamoxifen to exemestane after 2 to 3 years [45]. At a median follow-up of 5.1 years (60% of patients completed at least 5 years of follow-up), there was no significant difference in outcome between the two groups: DFS rates were 85% in the sequential arm and 86% in the exemestane-alone arm (Table 2) [55], and OS showed no significant difference, being 91% in both arms. In summary, there is no therapeutic gain in starting with tamoxifen and switching to an AI rather than starting with an AI upfront.

### Sequential tamoxifen after an aromatase inhibitor

The other BIG 1-98 crossover arm also addressed the question of tamoxifen after 2 to 3 years of letrozole. At a median follow-up of 8.0 years from random assignment, there was no significant difference between letrozole followed by tamoxifen versus letrozole alone (DFS hazard ratio = 1.06, *P* = 0.48; OS hazard ratio = 0.97, *P* = 0.79; Table 2) [54]. Therefore, the clinical implication is that a woman who wishes to switch from an AI to tamoxifen after 2 to 3 years because of side effects or for whatever reason can do so without adversely affecting outcome, at least for up to 5 years of treatment.

## Aromatase inhibitor toxicities and comparative toxicities with tamoxifen

AIs are associated with a higher incidence of musculoskeletal adverse events (MSAEs) (for example, myalgias and arthralgias), bone fractures, and decreased bone mineral density (BMD) [44,60-67]. In contrast, tamoxifen is associated with a higher incidence of thromboembolic and gynecological events (including endometrial cancer) [44,55,60,68-70]. Table 3 summarizes the incidence of treatment-related SAEs in the main adjuvant trials comparing AIs with tamoxifen.

**Table 3:** **Incidence of treatment-related serious adverse events in the main adjuvant aromatase inhibitor trials**[71]

**Study**	**Arms number**^**a**^**MSK: % (comparative *P *** **value)**^**b**^	**Arms number**^**a**^**BMD (T-score): % (comparative *P *** **value)**^**b**^	**Arms number**^**a**^**CV events: % (comparative *P *** **value)**	**Arms number**^**a**^**Gynae: % (comparative *P *** **value)**	**Arms number**^**a**^**Hot flashes : % (comparative *P *** **value)**
ATAC	A versus T 6,241	A versus T (5 years) 197	A versus T 6,186	A versus T (5 years) 6,186	A versus T (5 years) 6,186
	Arthralgia: 35.6 versus 29.4 (<0.0001)	LS: −6.1 versus +2.8 (<0.0001)	Ischemic CV event: 4.1 versus 3.4 (0.1)	Gynecologic event^d^: 3.0 versus 10.0 (<0.0001)	35.7 versus 40.9 (<0.0001)
			Ischemic CerebroV event: 2.0 versus 2.8 (0.03)	Vaginal bleeding. 5.4 versus 10.2 (<0.0001)	
	CTS: 3.0 versus 1.0 (<0.0001)	Hip: −7.2 versus +0.7 (<0.0001)	Venous TE event: 2.8 versus 4.5 (0.0004)	Vaginal discharge: 3.5 versus 13.2 (<0.0001)	
			DVT event: 1.6 versus 2.4 (0.02)	Reduced libido: 1.0 versus 0.4 (<0.0001)	
			CV death^c^: 2.0 versus 2.0 (NR)		
			CerebroV death^c^: 0.8 versus 0.9 (NR)		
			Hypercholesterolemia: 9.0 versus 3.0 (<0.0001)		
BIG 1-98	L versus T 8,028 (4,992)^a^	NA	L versus T 4,895	L versus T (6 years) 3,074	L versus T (6 years) 3,074
	Arthralgia: 20.0 versus 13.5 (<0.001)		Cardiac event: 5.5 versus 5.0 (0.48)	Vaginal bleeding: 5.1 versus 9.9 (<0.001)	37.7 versus 42.9 (NR)
	Myalgia: 7.1 versus 6.1 (0.19)		CerebroV accident or TIA: 1.4 versus 1.4 (0.90)	Night sweating: 15.6 versus 19.4 (NR)	
			TE event: 2.0 versus 3.8 (<0.001)		
			Hypercholesterolemia: 50.6 versus 24.6 (<0.001)		
TEAM	E versus T 9,779	E versus T (1 year) 161	E versus T 9,779	E versus T (2.75 years) 9,779	NA
	Arthralgia: 17.9 versus 9.2 (≤0.001)	LS: −2.8 versus +0.5 (0.0008)	Ischemic CV event/MI: 0.8 versus 0.7 (NR)	Endometrial hyperplasia: 0.0 versus 2.0 (<0.0001)	
				Vaginal hemorrhage: 1.6 versus 3.1 (<0.0001)	
		Hip: −2.2 versus +0.4 (0.04)			
				Vaginal discharge: 2.3 versus 6.8 (<0.0001)	
		FN: +0.3 versus −1.8 (0.414)		Vaginal infection: 0.7 versus 2.2 (<0.0001)	
MA.17	L versus placebo 5,187	L versus placebo 226	L versus placebo 5,187	L versus placebo (2.5 years) 5,187	L versus placebo 5,187
	Arthralgia: 25.0 versus 21.0 (<0.001)	LS: −5.4 versus −0.7 (0.008)	CV disease: 5.8 versus 5.6 (0.76)	Vaginal bleeding: 6 versus 8 (0.005)	58 versus 54 (0.003)
	Arthritis: 6.0 versus 5.0 (0.07)	Hip: −3.6 versus −0.7 (0.044)	MI: 0.3 versus 0.4 (NR) Stroke/TIA: 0.7 versus 0.6 (NR)	Vaginal dryness: 6 versus 5 (0.26)	
	Myalgia: 15.0 versus 12.0 (0.0041)		TE event: 0.4 versus 0.2 (NR) Hypercholesterolemia: 16 versus 16 (0.79)		

^a^Patients analyzed; ^b^values represent T-score change from baseline; ^c^data from 100-month analysis, n = 6,241; ^d^includes endometrial hyperplasia and neoplasia, cervical neoplasm, and enlarged uterine fibroids. A, anastrozole; ATAC, Arimidex, Tamoxifen, Alone or in Combination; BIG, Breast International Group; BMD, bone mineral density; CerebroV, cerebrovascular; CTS, carpal tunnel syndrome; CV, cardiovascular; DVT, deep vein thromboembolic; E, exemestane; FN, femoral neck; L, letrozole; LS, lumbar spine; MI, myocardial infarction; MSK, musculoskeletal; NA, not available; NR, not reported; T, tamoxifen; TE, thromboembolic; TEAM, Tamoxifen Exemestane Adjuvant Multinational; TIA, transient ischemic attack.

In the 10-year analysis of ATAC, fractures were more frequent during active treatment in the anastrozole versus tamoxifen arm (451 versus 351, OR = 1.33, *P* <0.0001) but were similar in the post-treatment follow-up period (110 versus 112, OR = 0.98, *P* = 0.9). Treatment-related SAEs were less common on anastrozole than on tamoxifen (223 versus 369, OR = 0.57, *P* <0.0001) but were similar after treatment completion (66 versus 78, OR = 0.84, *P* = 0.3). Anastrozole was associated with significantly less risk of endometrial cancer than tamoxifen (*P* = 0.02). No significant differences in non-BC deaths or in the incidence of other cancers were found between groups.

In the BIG 1-98 trial, the incidence of treatment discontinuation (13.6% versus 11.9% of patients on letrozole and tamoxifen, respectively, *P* = 0.08) as a result of an adverse event (AE) was greatest during the first 2 years of treatment and stabilized to an additional 1% to 2% per year for the remainder of the 5-year period. Endometrial cancer was diagnosed during treatment in 4 (0.2%) versus 11 (0.6%) patients on tamoxifen and letrozole, respectively. No significant difference between the two arms was observed regarding (non-breast) malignancies or deaths without prior cancer events.

In the TEAM study, generally, gynecological symptoms and PE occurred more frequently in the sequential treatment group than in the exemestane-only group and the opposite was seen regarding the incidence of MSAEs (50% versus 44%), osteoporosis, and fractures. Of note, the observation of increase in fractures with AIs has been made in trials which started over two decades ago, before bone health awareness, BMD testing, and bone agents (that is, bisphosphonates) were available. In the more contemporary MAP.3 breast cancer prevention trial comparing exemestane versus placebo, the absence of excess fragility fractures and total fractures and the ≥10% decreases in areal BMD in the exemestane group were reassuring [25,72]. Of note, similar baseline BMD in the two groups and the use of bisphosphonate therapy both before and during the study were reported in this study.

Several professional medical societies and organizations have published guidelines for the use of bisphosphonates in preventing and treating bone loss during AIs [21,22,73-75]. Therefore, the simple adherence to implemented standard medical practice (for example, bone health monitoring and vitamin D and calcium supplementation when appropriate) should largely obviate the fracture risk associated with AI use. Moreover, although AIs increase the rate of bone turnover and decrease bone density in post-menopausal women [76,77], these effects seem to diminish after completing AI therapy [78,79].

Regarding compliance, Cuzick and colleagues [80], in contrast with other investigators, noted better treatment adherence in patients experiencing vasomotor symptoms and joint symptoms. Toxicity may depend on the kind of patient reporting these symptoms and may be related to treatment compliance, which would explain improved treatment outcomes in these patients [81].

Interestingly, in 9,325 patients enrolled in the TEAM trial [82], patients with specific AEs - including vasomotor symptoms, MSAEs, and vulvo-vaginal symptoms - had significantly better DFS and OS at multivariate analysis and fewer distant metastases than patients reporting non-specific or no AEs (Table 4). Increasing numbers of specific AEs were also significantly associated with better survival outcomes. Similarly, a recent retrospective analysis of the BIG 1-98 trial suggests that the occurrence of arthralgia/myalgia/carpal tunnel symptoms at 3 and 12 months is associated with a significantly better DFS and BC-free interval irrespective of treatment (letrozole or tamoxifen) [83]. Certain specific AEs may be valuable predictors and biomarkers of treatment efficacy, although further prospective investigation is warranted.

**Table 4:** **Outcomes in relation to specific adverse events in the Tamoxifen Exemestane Adjuvant Multinational (TEAM) trial**[82]

**Outcome measure**	**AE**	**Number of AEs (yes versus no AE)**	**Hazard ratio**	**95% CI**	***P*** **value**
DFS	VMS	249 versus 837	0.731	0.618-0.866	<0.001
	MSAE	239 versus 847	0.826	0.694-0.982	0.030
	VVS	89 versus 997	0.769	0.585-1.01	0.058
	Overall	418 versus 668	0.735	0.632-0.855	<0.001
OS	VMS	147 versus 617	0.583	0.424-0.803	0.001
	MSAE	151 versus 613	0.811	0.654-1.005	0.055
	VVS	51 versus 713	0.570	0.391-0.831	0.003
	Overall	268 versus 496	0.680	0.565-0.819	<0.001
DM	VMS	165 versus 490	0.813	0.664-0.996	0.046
	MSAE	138 versus 517	0.749	0.601-0.934	0.010
	VVS	54 versus 601	0.687	0.435-1.085	0.107
	Overall	261 versus 394	0.783	0.651-0.942	0.010

*AE*, adverse event; *CI*, confidence interval; *DFS*, disease-free survival; *DM*, distant metastases; *MSAE*, musculoskeletal adverse event; *OS*, overall survival; *VMS*, vasomotor symptoms; *VVS*, vulvovaginal symptoms.

### Cognitive function with aromatase inhibitors

While many patients report ‘chemotherapy fog’ manifesting as a decrease in short-term memory during chemotherapy [84], less is known about the potential effects of endocrine therapy on cognitive function [71]. Breast Cancer Action conducted an online survey in 1,199 women on AIs and found that approximately 2% of the respondents experienced cognitive impairment and that nearly half (48%) reported ‘mental fuzziness’ which led only 3% to stop taking their AI [85]. Few of the large AI trials reported on cognitive function during treatment, so available data are limited.

In the ATAC trial, impairments in processing speed and verbal memory were reported in women on anastrozole as compared with healthy women [86]. A cross-sectional study in 31 post-menopausal women with early BC on anastrozole or tamoxifen for a minimum of 3 months [87] found significantly poorer verbal and visual learning and memory in the anastrozole versus tamoxifen group. These findings must be interpreted with caution because of the small sample size and use of a cross-sectional design.

A prospective analysis from BIG 1-98 showed that cognitive function was significantly better among patients on letrozole versus tamoxifen at the end of the 5-year treatment period [88]. A second analysis comparing the 5-year assessments with those collected about 1 year later showed a significant improvement in cognitive function of similar magnitude following completion of endocrine therapy in both groups [89].

A neuropsychological cross-sectional study from the TEAM trial evaluated the cognitive functioning during the first year’s treatment [90]. In the exemestane group (n = 62), 24% of patients reported reduced daily memory functioning compared with 6% of healthy controls, but there was no statistically significant difference between the two groups in any cognitive domain after 1 year of treatment [91]. Thus, the evidence of the effects of adjuvant endocrine therapy on cognitive function is limited and inconclusive, and further studies are required.

## Predictive factors of benefit from an aromatase inhibitor

As might be predicted, patients at highest risk based on the number of involved nodes, tumor grade, size, vascular invasion, and Ki67 gained most from an AI compared with tamoxifen [64,92,93], and a recent subset analysis of BIG 1-98 data also showed a more pronounced benefit of letrozole in invasive lobular versus invasive ductal carcinoma [94].

There is evidence from neoadjuvant studies suggesting that ER^+^HER2^+^ (human epidermal growth factor receptor) patients might respond better to AIs than to tamoxifen [95-97]. However, although data from the TEAM trial suggested a significant treatment-by-marker effect between AI/tamoxifen treatment and HER1, 2, and 3 expression in the 2.75 years prior to switching patients initially treated with tamoxifen to exemestane [98], this was not observed in the ATAC and BIG 1-98 trials. In these two trials, the HER2 status did not predict for benefit from an AI versus tamoxifen and patients with HER2-overexpressing or -amplified tumors were found to have a worse prognosis than HER2^−^ patients, regardless of whether they received tamoxifen or an AI [99,100]. Hence, the HER2 status is not considered a selection criterion for the most appropriate endocrine treatment.

## Extended adjuvant endocrine therapy

Women with HR^+^ early BC are at continuous risk of relapse up to 15 years after diagnosis, despite being on adjuvant endocrine therapy for around 5 years [13,101]. Several trials have addressed whether extended adjuvant endocrine therapy beyond 5 years reduces the risk of late recurrence.

### NCIC CTG MA.17/BIG 1-97

NCIC CTG MA.17/BIG 1-97 tested the effectiveness of 5 years of letrozole after completion of the standard 4 to 6 years of adjuvant tamoxifen and was the first phase III trial to demonstrate an OS advantage with an adjuvant AI [50]. At a median follow-up of 2.4 years, a highly significant reduction in the risk of recurrence was seen with letrozole versus placebo (DFS hazard = 0.57, *P* = 0.00008) [50]. Based on this, the safety monitoring committee recommended study unblinding, allowing patients in the control group to switch to letrozole (see below). At a median follow-up of 30 months, a relative reduction in recurrence risk of 42% occurred with letrozole [63]. Letrozole treatment significantly reduced the risk of distant metastases in both node-negative and -positive patients (*P* = 0.002) and significantly improved OS by 39% in node-positive patients compared with placebo (hazard ratio = 0.61, *P* = 0.04). A further ITT analysis of all outcomes, before and after unblinding, was performed at a median follow-up of 64 months (Table 3). Although 66% of women originally on placebo crossed over to letrozole, a 32% reduction in the hazard for a DFS event persisted for women originally randomly assigned to receive letrozole [102]. More recently, Jin and colleagues [56] conducted an analysis through an IPCW Cox model to adjust for the effects of treatment crossover, demonstrating at a median follow-up of 64 months that patients initially randomly assigned to receive letrozole had hazard ratios of 0.52, 0.51, and 0.61 for DFS, distant DFS (DDFS), and OS, respectively (all *P* <0.0001).

Exploratory and subgroup analyses of MA.17 showed that letrozole had similar benefits in older (>70 years, n = 1,323, 26%) versus younger (<60 years) patients without any increase in toxicity compared with placebo. Women who were pre-menopausal at diagnosis but who became post-menopausal during the initial 5 years (n = 889) experienced significantly greater benefit on letrozole (hazard ratio for DFS = 0.25) than older/post-menopausal women (n = 4,277) (hazard ratio = 0.69, *P* = 0.02 for interaction) [103]. Therefore, pre-menopausal BC patients who have become menopausal by the end of adjuvant tamoxifen also benefit significantly from extended adjuvant therapy.

The optimal duration of extended adjuvant endocrine therapy remains unclear. An exploratory analysis conducted by Ingle and colleagues [104] suggested that the hazard ratio continues to fall for DFS and DDFS but not for OS out to 48 months, indicating that the benefit of letrozole increases with longer exposure.

The 66% crossover rate in MA.17 from placebo to letrozole after unblinding offered a good opportunity to test whether delayed initiation of an AI could still be of any benefit [105]. At the time of trial unblinding, 1,579 women initially on placebo elected to receive letrozole and 804 women chose no further treatment. At a median follow-up of 5.3 years, a significant reduction in recurrence risk (adjusted hazard ratio = 0.37, *P* <0.0001) and a significant 61% improvement in DDFS were found in patients who switched to letrozole, although they had more adverse prognostic factors. These results suggest that therapy given more than 7 years after diagnosis can change the chronic relapsing behavior of HR^+^ BC. They also show that delayed letrozole commencement after stopping tamoxifen can still be of benefit.

### Other extended adjuvant therapy trials with aromatase inhibitors

Other trials have been conducted to investigate the role of extended adjuvant AI therapy (Table 1). In the ABCSG Trial 6a, HR^+^ post-menopausal patients who were disease-free after 5 years of adjuvant tamoxifen (with or without AG) were randomly assigned to 3 years of anastrozole or no further treatment [51]. With 856 patients and a median follow-up of 62.3 months, anastrozole further reduced the risk of a BC event (locoregional recurrence, distant recurrence, or contralateral BC) by 38% versus no further treatment (hazard ratio = 0.62, *P* = 0.031). There was no statistically significant difference in OS between the two arms.

NSABP-B33 investigated extended adjuvant therapy with exemestane in post-menopausal women with clinical T1-3N1M0 BC who were disease-free after 5 years of adjuvant tamoxifen [52]. This trial closed prematurely after the publication of the results of MA.17. At 30 months of median follow-up, ITT analysis showed a trend of improvement in 4-year DFS (91% versus 89%; relative risk 0.68; *P* = 0.07) and a statistically significant improvement in 4-year recurrence-free survival (96% versus 94%; RR = 0.44; *P* = 0.004).

The Adjuvant post-Tamoxifen Exemestane versus Nothing Applied trial compared exemestane versus observation after 5 years of previous tamoxifen [106]. This trial was prematurely closed after recruiting only 448 patients.

The data sets from these trials have been analyzed in an EBCTG meta-analysis [107]. At a median follow-up of 2.5 years, extended adjuvant AI treatment was associated with an absolute 2.9% decrease in BC recurrence (relative decrease of 43%, *P* <0.00001) and an absolute 0.5% decrease in BC mortality (relative decrease of 27%, *P* = 0.11). Of note, the authors emphasized that the magnitude of the effects seen on DFS and OS in these analyses is likely underestimated because of some crossover after unblinding.

### Ongoing studies

Several ongoing studies are investigating extended AIs in regard to optimal duration, intermittent versus continuous use, and benefit after AIs used during the first 5 years of therapy (Table 5) [108].

**Table 5:** Ongoing clinical trials of extended aromatase inhibitor therapy

**Study**	**Number**	**Population (treatment received pre-enrollment)**	**Arms at random assignment**	**Study number**
MA.17R	1,918	Prior 4.5-6 years of AI, with or without prior T^a^	L (5 years) versus placebo (5 years)	NCT00754845
		Completed AI ≤2 years prior random assignment		
SALSA	3,486	Any endocrine therapy (5 years)	A (5 years) versus A (2 years)	NCT00295620
LEAD	4,050	T (4-6 years)	L (5 years) versus L (2-3 years)	NCT01064635
DATA	1,900	T (2-3 years)	A (6 years) versus A (3 years)	NCT00301457
NSABP-B42	3,966	AI or T→AI^b^ (to 5 years)	L (5 years) versus placebo (5 years)	NCT00382070
SOLE	4,800	Any endocrine therapy^c^ (5 years)	L (5 years) versus intermittent^d^ L (5 years)	NCT00553410

^a^Including as part of MA.17. ^b^Tamoxifen must have been up to 3 years and may not have been given during years 4 and 5 of the 5 years of adjuvant hormonal therapy. ^c^Must have completed 4 to 6 years of prior selective estrogen receptor modulators or aromatase inhibitors (AIs), or a sequential combination of both. When calculating 4 to 6 years, neoadjuvant endocrine therapy should not be included. ^d^Intermittent: 48 months over 5 years: 4 × 9 months (9 months followed by 3-month treatment-free interval in years 1 to 4, at least 36 months) plus 1 × 12 months in years 5 at least 48 months. →, switch to; A, anastrozole; DATA, Different Durations of Anastrozole after Tamoxifen trial; L, letrozole; LEAD, Letrozole Adjuvant Therapy Duration trial; NSABP, National Surgical Adjuvant Breast and Bowel Project; SALSA, Secondary Adjuvant Long-term Study with Arimidex trial; SOLE, Study of Letrozole Extension trial; T, tamoxifen.

### Tamoxifen beyond 5 years

Results of relatively small trials assessing tamoxifen treatment for more than 5 years were inconclusive until recently [109-112]. The large Adjuvant Tamoxifen: Longer Against Shorter (ATLAS) trial addressed this uncertainty, and results on 6,846 women with ER^+^ disease randomly assigned to continue tamoxifen treatment to 10 years or not (control group) showed that 10 years of tamoxifen further reduced the risk of relapse (*P* = 0.002), BC mortality (*P* = 0.01), and all-cause mortality (*P* = 0.01) compared with 5 years [113]. Most of this benefit seemed to accrue late, and there were only modest reductions in recurrence rates during the 5 extra years of tamoxifen and a more impressive carryover benefit during the 5 years of follow-up after completion of 10 years of tamoxifen (Table 6). Furthermore, reduced mortality was apparent only after completion of 10 years of tamoxifen. Thus, the benefit of continuing tamoxifen for a further 5 years is the sum of the carryover benefit from the first 5 years and the sequential benefit of a further 5 years, giving a total estimated relapse risk reduction of 39% (*P* <0.0001) and risk reduction of BC mortality of 36% (*P* <0.0001). After completion of 10 years of tamoxifen, this estimated risk was reduced by 30% for relapse (2*P* = 0.01) and 48% for mortality (2*P* <0.0001), continuing for at least 5 years. These carryover benefits contribute substantially to the cumulative benefits of treatment, particularly because toxic effects occur mostly during the active treatment period. The most important AEs were an increased risk of endometrial cancer (RR = 1.74) and PE (1.87) after 10 years of treatment. Reassuringly, no increase was noted in stroke incidence, and a decrease in incidence of ischemic heart disease was noted (0.76). Overall the benefits of extended tamoxifen seemed to substantially outweigh the risks.

**Table 6:** Clinical trials testing tamoxifen beyond 5 years

**Study**	**Number**	**Population**	**Arms**	**Disease-free survival hazard ratio (95% CI)**	**Overall survival hazard ratio (95% CI)**
ATLAS [113] Open-label	6,846^a^	Pre- and post-menopausal women with ER^+^ EBC who already received T for 5 years (in the context of ATLAS trial total number = 12,894)	T for additional 5 years (10 years) versus stop T (5 years)	5-9 years RR 0.90 (0.79-1.02) *P* = 0.10 > 10 years RR 0.75 (0.62-0.90) *P* = 0.01	BC mortality: 5-9 years RR 0.97 (0.79-1.18) *P* = 0.74 BC mortality: >10 years RR 0.71 (0.58-0.88) *P* = 0.002
				All years log-rank = 0.002	
				Absolute reduction at years 15: 3.7%	
aTToM [114]^b^ Open-label	6,953	Invasive EBC who had already been taking T for 5 years. 2,755 ER^+^ (39%) and 4,198 ER untested (61%) (estimated 80% ER^+^ if status unknown)	T for additional 5 years versus no further treatment	RR 0.85 (0.76-0.95) *P* = 0.003	BC mortality: 5-9 years RR 1.08 (0.85-1.38)
				Absolute reduction 4%	BC mortality: >10 years RR 0.75 (0.63-0.90) *P* = 0.007 BC mortality: all years RR 0.88 (0.74-1.03) *P* = 0.1
Pooled analysis ATLAS + aTToM [114]^b^	17,477	10,543^c^ ER^+^ from ATLAS plus 6,934 ER^+^ from aTTom	T 10 versus 5 years	NA	BC mortality: 5-9 years RR 0.97 (0.84-1.15)
					BC mortality: >10 years RR 0.75 (0.65-0.86) *P* = 0.00004
					BC mortality: all years RR 0.85 (0.77-0.94) *P* = 0.001

^a^Analysis of estrogen receptor-positive (ER^+^) patients only; ^b^figures are derived from the abstract [114] and the presentation at American Society of Clinical Oncology meeting 2013 available online; ^c^IPCW (inverse probability of censoring weighted) estimate of the effect in ER^+^. ATLAS, Adjuvant Tamoxifen: Longer Against Shorter; aTToM, Adjuvant Tamoxifen-To Offer More?; BC, breast cancer; CI, confidence interval; EBC, early breast cancer; RR, rate ratio; T, tamoxifen.

Likewise, the Adjuvant Tamoxifen-To Offer More? (aTTom) trial randomly assigned 6,953 UK women in remission after 5 years of tamoxifen to 5 more years of tamoxifen or to stop (Table 6) [114]. The compliance rate was 75% in the 10-year tamoxifen study arm. The BC recurrence rates were 16.7% in the 10-year study group and 19.3% in the 5-year study group. Similarly to the ATLAS trial, there was a time-dependent reduced risk of recurrence with 10 years of tamoxifen with RRs of 0.99 during years 5 to 6, 0.84 during years 7 to 9, and 0.75 subsequently. Longer treatment also reduced BC mortality in a time-dependent fashion with RRs of 1.03 during years 5 to 9 and 0.77 later and overall mortality RRs of 1.05 during years 5 to 9 and 0.86 later. Non-BC mortality was little affected (457 versus 467 deaths; RR = 0.94). The most serious AE of long-term tamoxifen was an increase in endometrial cancer risk: there were 102 versus 45 endometrial cancers (RR = 2.20, *P* <0.0001) with 37 (1.1%) versus 20 (0.6%) deaths (absolute hazard 0.5%, *P* = 0.02). The pooled analysis of the UK aTTom and the international ATLAS trials showed enhanced significance of recurrence (*P* <0.0001), BC mortality (*P* = 0.002), and OS (*P* = 0.005) benefits [114].

In conclusion, in ER^+^ disease, continuing tamoxifen to year 10 rather than just to year 5 produces further reductions in recurrence, from year 7 onward, and BC mortality after year 10. Taken together with the reduction in BC deaths seen in trials of 5 years of tamoxifen versus none, these results indicate that adjuvant tamoxifen for 10 years, compared with no tamoxifen, reduces BC mortality by about one third in the first 10 years after diagnosis and by half subsequently. No significant heterogeneity was observed in the proportional risk reduction with respect to patient or tumor characteristics or site of first relapse.

The important questions of which patients really benefit and whether extended adjuvant endocrine therapy should be with tamoxifen or an AI in post-menopausal women currently remain unanswered. Active research is currently ongoing on molecular features and gene expression scores combined with standard clinico-pathological criteria to tailor extended endocrine therapy [115].

## Conclusions

Adjuvant endocrine therapy significantly reduces the risk of recurrence and death in women with early HR^+^ BC. In pre-menopausal women, tamoxifen and OvS are of similar benefit. Currently there is no strong evidence that combined treatment is better than either alone, but results of two major trials addressing the value of additional OvS are awaited. In post-menopausal women, AIs are significantly more effective than tamoxifen in preventing recurrence but so far only letrozole has been shown to have survival benefit. For women at only low or moderate risk, there is little difference in efficacy between the two treatments.

Extended adjuvant endocrine therapy with an AI (post-menopausal) or tamoxifen beyond an initial 5 years of tamoxifen further reduces the risk of relapse. The relative merits of these two approaches and the selection of patients requiring long-term endocrine therapy are now important questions requiring further research, as is the important issue of the optimum duration of an AI if started upfront rather than after tamoxifen.

## Abbreviations

ABCSG: Austrian breast and colorectal cancer study group; AE: Adverse event; AG: Aminoglutethimide; AI: Aromatase inhibitor; ATAC: Arimidex, tamoxifen, alone or in combination; ATLAS: Adjuvant tamoxifen: longer against shorter; aTTom: Adjuvant tamoxifen-to offer more?; BC: Breast cancer; BIG: Breast international group; BMD: Bone mineral density; CYP2D6: Cytochrome P450 2D6; DDFS: Distant disease-free survival; DFS: Disease-free survival; EBCTCG: Early breast cancer trialists’ collaborative group; ER: Estrogen receptor; HER: Human epidermal growth factor receptor; HR: Hormone receptor; IES: Intergroup exemestane study; IPCW: Inverse probability of censoring weighted; ITT: Intention to treat; LHRH: Luteinizing hormone-releasing hormone; MSAE: Musculoskeletal adverse event; NSABP: National surgical adjuvant breast and bowel project; OS: Overall survival; OvA: Ovarian ablation; OvS: Ovarian suppression; PE: Pulmonary embolus; PgR: Progesterone receptor; RR: Rate ratio; SAE: Serious adverse effect; SOFT: Suppression of ovarian function trial; TEAM: Tamoxifen exemestane adjuvant multinational; TEXT: Tamoxifen and exemestane trial; TTDR: Time to distant recurrence; TTR: Time to recurrence.

## Competing interests

The authors declare that they have no competing interests.
